# Magnetoelectric Membrane Filters of Poly(vinylidene fluoride)/Cobalt Ferrite Oxide for Effective Capturing of Particulate Matter

**DOI:** 10.3390/polym12112601

**Published:** 2020-11-05

**Authors:** Kyujin Ko, Su-Chul Yang

**Affiliations:** Department of Chemical Engineering (BK21 FOUR), Dong-A University, Busan 49315, Korea; rbwls0096@gmail.com

**Keywords:** magnetoelectric membrane filters, poly(vinylidene fluoride), cobalt ferrite oxide, capturing efficiency, particulate matter

## Abstract

In the last decade, particulate matter (PM) has gradually become a serious public health issue due to its harmful impact on the human body. In this study, we report a novel filtration system for high PM capturing, based on the magnetoelectric (ME) effect that induces an effective surface charge in membrane filters. To elucidate the ME effect on PM capturing, we prepared electrospun poly(vinylidene fluoride)(PVDF)/CoFe_2_O_4_(CFO) membranes and investigated their PM capturing efficiency. After electrical poling under a high electric field of 10 kV/mm, PM-capturing efficiencies of the poled-PVDF/CFO membrane filters were improved with carbon/fluorine(C/F) molar ratios of C/F = 4.81 under *H*_dc_ = 0 and C/F = 7.01 under *H*_dc_ = 700 Oe, respectively. The result illustrates that electrical poling and a dc magnetic field could, respectively, enhance the surface charge of the membrane filters through (i) a strong beta-phase alignment in PVDF (poling effect) and (ii) an efficient shape change of PVDF/CFO membranes (magnetostriction effect). The diffusion rate of a water droplet on the PVDF/CFO membrane surface is reduced from 0.23 to 0.05 cm^2^/s by covering the membrane surface with PM. Consequently, the PM capturing efficiency is dramatically improved up to 175% from ME membranes with the poling process and applying a magnetic field. Furthermore, the PM was successfully captured on the prototype real mask derived from the magnetoelectric effect induced by a permanent magnet with a diameter of 2 cm without any external power.

## 1. Introduction

Membrane technology has been implicated in a variety of environmental fields, such as water purification, gas barrier, and particulate matter (PM) filters [1,2,3,4,5,6,7]. In particular, the harmful effects of PM in the air on the human circulatory system have been recently reported [8,9,10,11,12,13,14]. PM is composed of NO_3_^−^, SO_4_^2−^, and CO_x_ as a mixture of solid particles and liquid droplets [15,16,17,18,19,20]. Ambient PM is classified as PM_10_ and PM_2.5_ with aerodynamic diameters below 10 and 2.5 μm, respectively [21,22,23,24,25]. In particular, PM_2.5_ easily reaches the human lungs and bronchi, causing serious respiratory and cardiovascular diseases [26,27,28,29,30]. The World Health Organization has designated PM_2.5_ as a serious carcinogen with strict exposure limits [31].

Many research groups have developed effective PM removal systems with a low-pressure drop via different routes. Zhang et al. designed an ultrathin dust filter system made of poly(m-phenylene isophthalamide) nanofiber/two-dimensional nanonets to efficiently capture PM without affecting air permeability [32]. Li et al. and Jung et al. used electrical poling on polyacrylonitrile nanofibers and reduced graphene oxide membranes, respectively, using clamped filters with two metal meshes to induce stronger surface charges in the filters [20,33]. Jeong et al. developed high-efficiency (>99.999%) air filters made of Ag nanowires on a nylon mesh, with the applied voltage between 2.5 and 10 V [34]. Some research groups have investigated the dipole moments of polyvinylidene fluoride (2.1 D), polyvinylpyrrolidone (2.3 D), polyvinyl alcohol (3.6 D), and polycarbonate (4.1 D), as a dipole-dipole moment or an induced-dipole force in the polymers could actively capture PM on the polymer surfaces [35,36,37]. Even though functional membrane filters have been gradually developed, there are still critical limitations of low surface charge and applying external power with wire systems.

In this study, to induce high surface charge under a self-powered wireless system, we designed a novel PM filtration system using a magnetoelectric (ME) effect, which can induce a strong surface charge in the membrane filters via an induced strain change in polymer composites of poly(vinylidene fluoride) (PVDF) and cobalt ferrite oxide (CoFe_2_O_4_, CFO). The ME effect is defined as an induced electrical polarization by applying a magnetic field, and feasible ME properties can be obtained in a two-phase system consisting of piezoelectric and magnetostrictive phases [38,39]. Song et al. investigated high saturated magnetization of CFO in respect to crystal-dependent coercivity [40]. So far, two-phase ME composites have been studied to achieve high ME voltage for practical applications such as energy harvesters, magnetic-/electric-sensors, or transducers [41,42,43]. In 2016, Rodzinski et al. reported a new drug delivery system using two-phase ME materials with a core-shell structure to actively target cancer tumors, and efficiently release treatment drugs [44]. In the ME drug delivery system, the main concept was to induce sufficient surface charge on the core-shell drugs by applying a dc magnetic field (*H*_dc_) for actively targeting the tumor cells [45,46,47,48]. Based on previous studies on the surface charge control of ME materials, as an innovation in the environmental field, we developed a two-phase ME membrane with a strong surface charge for effective PM filtration. Under an applied dc magnetic field, induced magnetostriction in the magnetostrictive phase of CFO can be transferred to the piezoelectric phase of PVDF, causing a strong electrical charge on the surface of the ME membrane filters given by the following Equation (1).

(1) $$\mathrm{Surface}\;\mathrm{charge}\;\mathrm{of}\;\mathrm{ME}\;\mathrm{membranes}={\left(\frac{\mathrm{strain}}{\mathrm{magnetic}\;\mathrm{field}}\right)}_{\mathrm{CFO}}\times {\left(\frac{\mathrm{electric}\;}{\mathrm{strachargein}}\right)}_{\mathrm{PVDF}}$$

**Figure 1:**
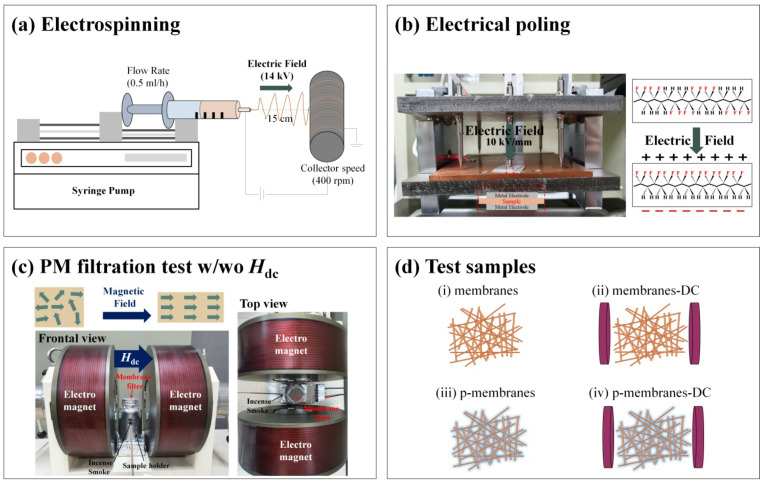
(**a**) Schematic illustration of the electrospinning process for the fabrication of poly(vinylidene fluoride)/CoFe_2_O_4_ (PVDF/CFO) membranes, (**b**) poling process for beta-phase alignment in PVDF, (**c**) setup for particulate matter (PM) filtration test, and (**d**) four types of test samples; (i) PVDF/CFO membranes under *H*_dc_ = 0 Oe (membranes), (ii) PVDF/CFO membranes under *H*_dc_ = 700 Oe (membranes-DC), (iii) poled-PVDF/CFO membranes under *H*_dc_ = 0 Oe (p-membranes), and (iv) poled-PVDF/CFO membranes under *H*_dc_ = 700 Oe (p-membranes-DC).

To fabricate the two-phase ME membrane filters, the CFO nanoparticles were hydrothermally synthesized with a primary particle size of 20 nm, and then the porous membranes of PVDF/CFO were prepared using an electrospinning method as shown in Figure 1a. The ME membrane filter systems were evaluated based on PM capturing efficiency with respect to poling effect and magnetostriction effect, which were adjusted by applying an electrical poling of 10 kV/mm and a dc magnetic field of 700 Oe to the PVDF/CFO membranes as shown in Figure 1b,c. To investigate the poling and magnetostriction effects on PM capture, four types of filtration test were conducted as shown in Figure 1d: (i) unpoled membranes under zero dc magnetic field, *H*_dc_ = 0 Oe (membranes), (ii) unpoled membranes under a dc magnetic field of *H*_dc_ = 700 Oe (membranes-DC), (iii) poled-membranes under zero dc magnetic field, *H*_dc_ = 0 Oe (p-membranes), and (iv) poled-membranes under a dc magnetic field of *H*_dc_ = 700 Oe (p-membranes-DC). Both effects on the membrane surface charge were clearly revealed from comprehensive results illustrating active PM capture in the ME filtration system.

## 2. Experimental

### 2.1. Materials and Reagents

Iron (III) nitrate nonahydrate ((Fe(NO_3_)_3_)∙9H_2_O, ≥98%), Cobalt (II) nitrate hexahydrate ((Co(NO_3_)_2_)·6H_2_O, ≥98%), Sodium Hydroxide (NaOH, ≥98%), Poly(vinylidene fluoride) pellets (PVDF, *M*_w_ = ~530,000 g mol^−1^), N,N dimethylformamide (HCON(CH_3_)_2_, DMF, 99.8%), and Acetone (CH_3_COCH_3_, ≥99.9%) were purchased from Sigma-Aldrich, Yongjin, Korea.

### 2.2. Synthesis of CFO Nanoparticles

As a magnetostrictive phase, CFO nanoparticles were hydrothermally synthesized [49]. A precursor solution was prepared by dissolving Iron (III) nitrate nonahydrate of 2 mmol and Cobalt (II) nitrate hexahydrate of 1 mmol in distilled water of 20 mL. Then, 10 M sodium hydroxide solution was added to the precursor solution with stirring for 1 h to adjust to pH 13. The mixed dark brown solution was transferred into a 50 mL Teflon-lined stainless-steel autoclave. Then, hydrothermal synthesis was conducted at 200 °C for 2 h. The prepared CFO nanoparticles were finally washed with ethanol.

### 2.3. Fabrication of PVDF/CFO Membranes

Fifteen wt % PVDF pellets were completely dissolved in a solvent consisting of 50/50 (*v/v*) DMF and acetone at 60 °C for 4 h. The CFO nanoparticles in a colloidal acetone solution were mixed with the PVDF solution by vigorous stirring at 700 rpm for 10 h. Porous membranes of PVDF/CFO were then fabricated at room temperature under an optimized electrospinning condition with an applied electric field of 14 kV. The distance between needle and collector was approximately 15 cm, and a flow rate of 0.5 mL/h [50]. The electrical poling of the PVDF/CFO membranes was conducted at 10 kV/mm for 1 h.

### 2.4. Characterization

Crystalline structures of CFO, PVDF, PVDF/CFO were investigated by X-ray diffraction (XRD, Miniflex600, RIGAKU Ltd., Tokyo, Japan) with CuK_α_ (λ = 1.5406 Å) radiation. Morphology and elementary composition of CFO, PVDF, PVDF/CFO, and PM were confirmed by scanning electron microscopy (SEM, JEOL-6700F, JEOL Ltd., Kyoto, Japan), transmission electron microscopy (TEM, Talos F200X, Thermo Scientific Ltd., Waltham, MA, USA) and energy dispersive spectrometry (EDS, JEOL-6700F, JEOL Ltd., Kyoto, Japan). Post PM filtration, the diffusion rate of a water droplet on the PVDF/CFO membranes was measured using a contact angle analyzer (FM-40, KRUSS Ltd., Hamburg, Germany). Compositions of captured PM on the PVDF/CFO membranes were characterized by Fourier transform infrared spectroscopy (FT-IR, Nicolet-380, Thermo Scientific Ltd., Waltham, MA, USA) and X-ray photoelectron spectroscopy (XPS, K-ALPHA+XPS System, Thermo Scientific Ltd., Waltham, MA, USA) with monochromatic Al Kα (*hv* = 1486.6 eV) radiation. Piezoelectric output voltage of the PVDF/CFO membranes was measured using a source meter (Keithley 2450, Tektronix Inc., Beaverton, OR, USA) by fingers tapping on a soft substrate.

### 2.5. Investigation of PM Capturing Efficiency

The PM capturing efficiency of the PVDF/CFO membranes was characterized by a comprehensive analysis including SEM, EDS, contact angle, FT-IR spectroscopy, and XPS data, after a filtration test of incense smoke. Membrane filters were placed on the top of a sample holder which has a window to load fixed incense into the holder as shown in Figure 1c. The distance between the side electromagnets is 5 cm and a constant dc magnetic field (*H*_dc_) was applied to induce magnetostriction of the CFO nanoparticles. Under two applied conditions of *H*_dc_ = 0 Oe and *H*_dc_ = 700 Oe, filtration tests were conducted by burning incense at 0.35 g/h for 30 min.

## 3. Results and Discussion

**Figure 2:**
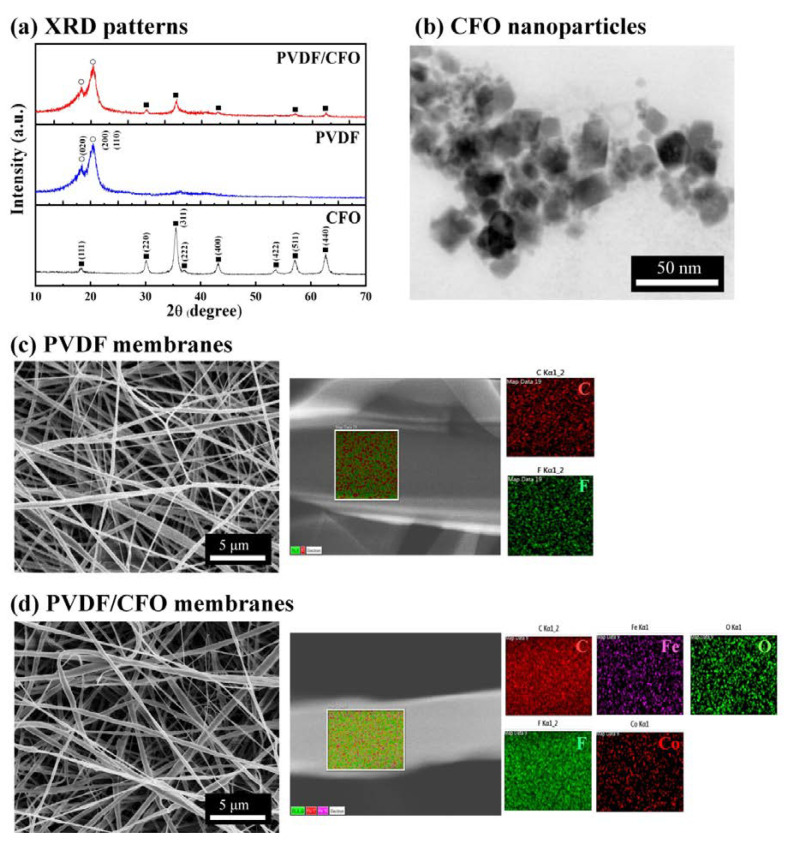
(**a**) X-ray diffraction (XRD) patterns of CFO nanoparticles, PVDF membranes, and PVDF/CFO membranes, (**b**) a transmission electron microscope (TEM) image of CFO nanoparticles, (**c**) a scanning electron microscope (SEM) image with energy dispersive spectrometer (EDS) analysis of PVDF membranes, and (**d**) an SEM image with EDS analysis of PVDF/CFO membranes.

Figure 2a shows the X-ray diffraction (XRD) patterns of CFO nanoparticles, PVDF membranes, and PVDF/CFO membranes. The CFO nanoparticles were confirmed to have a clear spinel structure of CoFe_2_O_4_ with representative XRD peaks of (111), (220), (311), (222), (400), (422), (511) and (440) [51]. The PVDF membranes were found to exhibit XRD peaks of (020), (200), and (110) illustrating the coexistence of the α- and β-phases [52,53]. The ME membranes of PVDF/CFO were successfully prepared without any secondary phases [50,54]. From Figure 2b, the primary particle size of the CFO nanoparticles was confirmed to be ~20 nm; therefore, the magnetostrictive CFO nanoparticles could be well embedded in the piezoelectric PVDF fibers. The PVDF membranes consisting of carbon and fluorine were formed as stacked slender fibers of irregular diameters under 500 nm as shown in Figure 2c. The PVDF/CFO membranes consisting of carbon, fluorine, cobalt, ferrite, and oxide exhibited a form similar to the PVDF membranes as shown in Figure 2d. The EDS images shown in Figure 2d reveal that CFO nanoparticles were homogeneously dispersed in the PVDF fibers.

**Figure 3:**
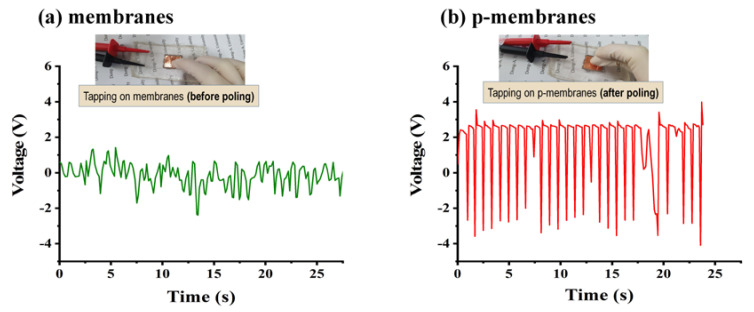
Tapping-mode piezoelectric output voltages of (**a**) membranes and (**b**) p-membranes.

As shown in Figure 3, the piezoelectric output voltage of the PVDF/CFO membranes was investigated to verify the induced surface charge of the membranes due to electrical poling. With a constant finger tapping the membranes, p-membranes were found to exhibit a clear piezoelectric voltage response between −4 and 2 V due to an induced electrical polarization on the surface of the membranes. Further, to enhance the PM capture efficiency, the surface charge of the p-membranes was amplified via a strain transfer of the magnetostriction to the piezoelectric phase [31,55,56,57].

**Figure 4:**
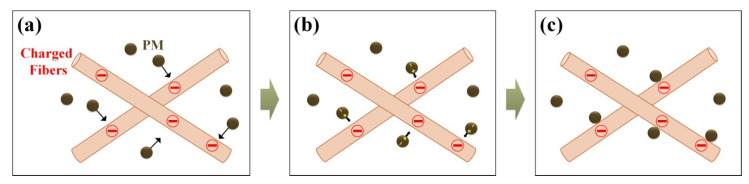
Schematic diagram of a PM-capturing mechanism with three main steps: (**a**) neutral condition of PM charge, (**b**) partially induced PM charge by London dispersion force, and (**c**) PM capture by an electrophoretic force.

According to previous studies on dust capturing systems using the surface charge of membrane filters, there are three steps in a capturing mechanism that can apply to the ME filtration system as shown in Figure 4 [58]. The main mechanism of PM capture was derived from comparatively weak intermolecular forces of Van der Waals force induced by the dipole of polymer and PM molecules [59,60]. At the first step, the PM charge is neutral when the PM and membrane fibers are far apart. At the second step, as they come closer, momentarily, dipoles of PM are formed by London dispersion forces causing a temporary dipole moment in nonpolar molecules as described by the Equation (2) [34]. The London dispersion force is a weak intermolecular force with a temporary dipole as Van der Waals force when two electrons are placed closer together [61]. At the final step, the charged PM is attracted to the charged membrane fibers by an electrophoretic force [62].

(2) $$F=\;\frac{\pi^{2}q_{0}^{2}\lambda }{12x^{2}}\left(\frac{d_{1}d_{2}}{d_{1}+d_{2}}\right)$$

where F = attractive force, dynes; x = distance of separation, cm; λ = London-van der Waals constant; $q_{0}$ = no. of atoms in one cm^3^ of the substance; $d_{1}d_{2}$ = diameters of spherical particles, cm; $\pi^{2}q_{0}^{2}\lambda$ = 10^−12^ as given by Hamaker.

**Figure 5:**
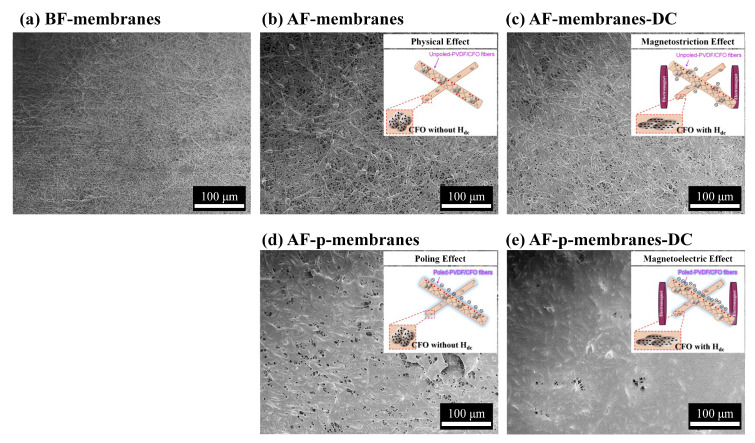
SEM images of (**a**) PVDF/CFO membranes before PM filtration (BF-membranes), (**b**) PVDF/CFO membranes after PM filtration under *H*_dc_ = 0 Oe (AF-membranes), (**c**) PVDF/CFO membranes after PM filtration under *H*_dc_ = 700 Oe (AF-membranes-DC), (**d**) poled-PVDF/CFO membranes after PM filtration under *H*_dc_ = 0 Oe (AF-p-membranes), and (**e**) poled-PVDF/CFO membranes after PM filtration under *H*_dc_ = 700 Oe (AF-p-membranes-DC).

As shown in Figure 5, the surface morphology of the PVDF/CFO membranes was investigated to visibly compare the amount of PM captured on the membrane filters: (a) PVDF/CFO membranes before PM filtration (BF-membranes), (b) PVDF/CFO membranes after PM filtration under *H*_dc_ = 0 Oe (AF-membranes), (c) PVDF/CFO membranes after PM filtration under *H*_dc_ = 700 Oe (AF-membranes-DC), (d) poled-PVDF/CFO membranes after PM filtration under *H*_dc_ = 0 Oe (AF-p-membranes), and (e) poled-PVDF/CFO membranes after PM filtration under *H*_dc_ = 700 Oe (AF-p-membranes-DC). As shown in Figure 5a, the membranes were found to present a porous structure between stacked fibers without any captured PM. As shown in Figure 5b, after the PM filtration, pores of the AF-membranes were covered with the PM of incense smoke; however, there are still remnant vacant pores due to insufficient PM capture via the physical capturing mechanism. As shown in Figure 5c, on applying *H*_dc_ of 700 Oe, the magnetostriction of the CFO nanoparticles could be transferred to PVDF, increasing the amount of PM captured on the AF-membranes-DC due to induced surface charge of membranes given by an effective shape change of the PVDF [55,56,57]. As shown in Figure 5d, after the electrical poling of 10 kV/mm, the surface of the AF-p-membranes was fully covered by the PM owing to the strengthening of the surface charge by the beta-phase alignment in PVDF along the poling direction [63,64,65]. As shown in Figure 5e, on applying *H*_dc_ of 700 Oe, the PM of the incense smoke was fully trapped, confirmed by the dense PM layer on the surface of the AF-p-membranes-DC. This could be attributed to the inducing of a strong surface charge on the AF-p-membranes-DC due to a strong shape change of the electrically polarized-piezoelectric phase.

**Figure 6:**
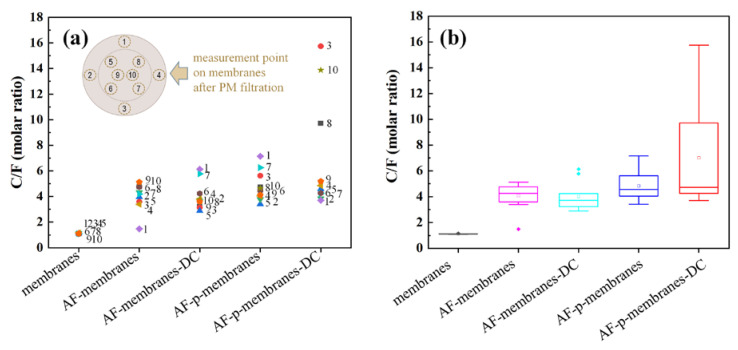
PM capturing efficiency of PVDF/CFO membranes based on Carbon/Fluorine (C/F) molar ratio: (**a**) region-dependent PM capture, and (**b**) the mean value of PM capture.

To quantitatively compare the PM capturing efficiency of the ME membrane filters, EDS mapping was systematically conducted on the whole area of the membranes with a mapping range of 2.5 mm^2^ per point as shown in the inset of Figure 6a. Before the PM filtration, the PVDF/CFO membranes had a Carbon/Fluorine (C/F) molar ratio of 1 with a narrow variation because the molecular formula of vinylidene fluoride is C_2_H_2_F_2_. After the PM filtration, the C/F molar ratio in the AF-membranes increased with a minor regional difference, which implied that some amount of PM was captured at the center of the membranes. On applying the *H*_dc_, compared to the AF-membranes, minimal and maximal C/F molar ratios simultaneously increased without any regional difference due to the magnetostriction effect in the AF-membranes-DC. After the electrical poling, compared to the AF-membranes-DC, a slight improvement in C/F molar ratio with a similar variation was observed in the AF-p-membranes. On applying the *H*_dc_ to the AF-p-membranes-DC, a drastically enhanced C/F molar ratio was achieved in the ME filtration system involving poling and magnetostriction effects. Even though the variation in the C/F molar ratio is in a wide range from 3.7 to 15.7, it clearly illustrates that the ME membranes with a high surface charge have a strong attractive force to efficiently capture PM. As shown in Figure 6b, the mean values of C/F molar ratio were investigated with an interquartile range to obtain a reliability of the point results. The unpoled samples of AF-membranes and AF-membranes-DC showed mean C/F molar ratios of 4.05 and 4.02, respectively, with a similar interquartile range. On the other hand, after electrical poling, mean C/F molar ratios of AF-p-membranes and AF-p-membranes-DC increased up to 4.81 and 7.01, respectively. The C/F molar ratio of PVDF/CFO membranes was compared in Table 1. Compared to the AF-membranes under the physical capturing system, the AF-p-membranes-DC exhibited an enhanced capturing efficiency of 175% under the magnetoelectrical capturing system.

**Figure 7:**
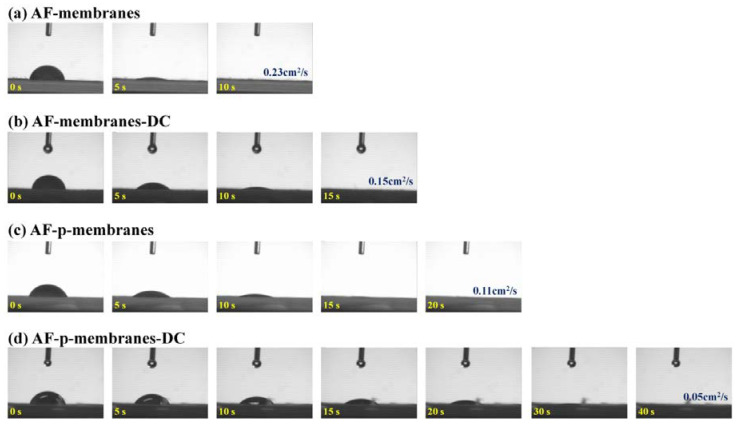
Diffusion rate of a water droplet on the PVDF/CFO membranes: (**a**) AF-membranes with 0.23 cm^2^/s, (**b**) AF-membranes-DC with 0.15 cm^2^/s, (**c**) AF-p-membranes with 0.11 cm^2^/s, and (**d**) AF-p-membranes-DC with 0.05 cm^2^/s.

As shown in Figure 7, the diffusion rate of a water droplet on the surface of PVDF/CFO membranes, which is related to the membrane hydrophobicity and PM layer density, was measured to analyze the PM coverage on the membranes. An increase in the captured PM, consisting of mainly carbon, resulted in a decrease in the diffusion rate due to the strong hydrophobicity and a dense PM layer on the membrane surface. With a high PM capture, pores of the PVDF/CFO membranes were covered with PM to prevent the diffusion of the droplet into the membranes. The diffusion rate was found to be 0.23, 0.15, 0.11, and 0.05 cm^2^/s on the AF-membranes, AF-membranes-DC, AF-p-membranes, and AF-p-membranes-DC, respectively. The five times slower diffusion rate for the AF-p-membranes-DC implies enhanced membrane hydrophobicity and a high PM layer density. The diffusion rate of a water droplet on the PVDF/CFO membranes was compared in Table 2.

**Figure 8:**
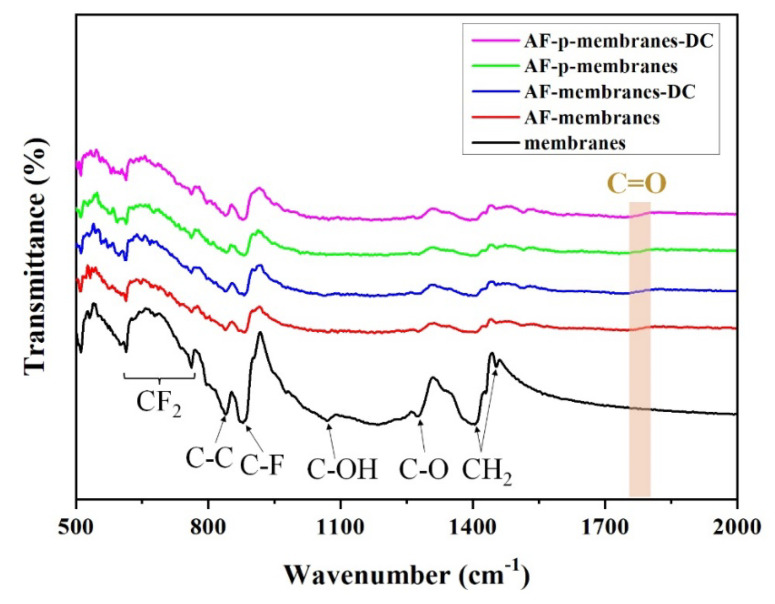
Fourier transform infrared spectroscopy (FT-IR) spectra of the PVDF/CFO membranes.

The chemical bonds in the PVDF/CFO membranes were investigated by Fourier transform infrared (FT-IR) spectroscopy before and after PM filtration. As shown in Figure 8, the membranes without any captured PM showed corresponding peaks of CF_2_ stretching vibrations at 599.78, 613.28, and 761.78 cm^−1^, C–C asymmetrical stretching vibration at 840 cm^−1^, C–F stretching vibration at 882 cm^−1^, C–OH stretching and C–O stretching vibrations at 1066.49 and 1280.56 cm^−1^, respectively, CH_2_ wagging vibration at 1404 cm^−1^, and C=O ester group vibration at 1740 cm^−1^ [66,67,68,69,70]. After the PM filtration, all PVDF/CFO membranes exhibited a decreased transmittance with a noticeable peak of C=O ester group vibration at 1730 cm^−1^, which might be a chemical bond in the captured PM [17,71].

**Figure 9:**
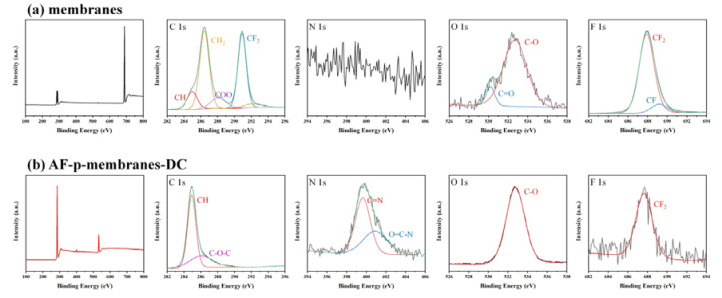
X-ray photoelectron spectroscopy (XPS) of (**a**) membranes and (**b**) AF-p-membranes-DC.

X-ray photoelectron spectroscopy (XPS) analysis was conducted to analyze the chemical binding characteristics in the captured PM on the PVDF/CFO membranes. As shown in Figure 9a, the membranes before the PM filtration showed a CH bond at 284.98 eV, a CH_2_ bond at 286.48 eV, a COO bond at 287.98 eV, a CF_2_ bond at 290.88 eV, a C=O bond at 530.18 eV, a C-O bond at 532.68 eV, a CF_2_ bond at 687.88 eV, and a CF bond at 689.18 eV. After the PM filtration, the CH_2_ bond at 286.48 and CF_2_ bond at 290.88 eV representing PVDF clearly disappeared, while the C=N bond at 399.68 and the O=C–N bond at 400.88 eV, characteristic of PM, appeared as shown in Figure 9b [49,72,73,74,75,76,77,78].

**Figure 10:**
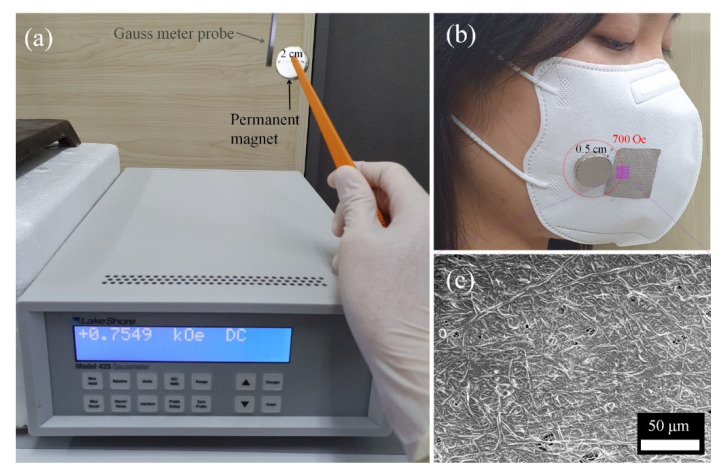
(**a**) Magnetic field measurement for a permanent magnet, (**b**) prototype mask using ME membrane filters, and (**c**) SEM images of ME membranes after PM filtration.

To confirm the PM capturing effect of the ME membranes in a daily life condition, a PM filtration test using a prototype mask was conducted as shown in Figure 10. From Figure 10a, a small permanent magnet exhibited around 700 ± 50 Oe at 0.5 cm distance from the Gauss meter probe. As shown in Figure 10b, with an attachment of the permanent magnet on the prototype mask, a filtration test of incense smoke was successfully performed. As shown in Figure 10c, it is highlighted that the ME membranes effectively captured incense smoke PM by just attaching the small magnet as a wireless and self-powered filtration system.

## 4. Conclusions

In this study, we reported a novel filtration system for particulate matter (PM) based on the ME effect in PVDF/CFO membranes. Efficient PM capture was achieved by applying electrical poling of 10 kV/mm and the dc magnetic field of 700 Oe to the PVDF/CFO membranes. With the poling and magnetostriction effects in the ME system, the surface charge of the membranes could be maximized resulting in a high PM capturing efficiency. The investigation of C/F molar ratio showed that, compared to a physical capturing system, the PM capture in the ME system significantly increased by up to 175%. The high PM capture was further confirmed by the five times slower diffusion rate of a water droplet on the surface of ME membranes. We believe that these ME membranes with wireless and self-powered configurations present a significant opportunity in the field of PM filtration. The prototype ME membrane filters successfully captured PM on the surface by inducing surface charge using only a permanent small magnet. Furthermore, for reusable PM membranes, non-contact PM elimination is possible in the ME filter system via high-frequency ac magnetic vibration from CFO particles.

## Figures and Tables

**Table 1:** C/F molar ratio of the PVDF/CFO membranes.

Membranes	C/F (Molar Ratio)		
	Minimum	Maximum	Mean
Membranes	1.09	1.16	1.12
AF-membranes	1.48	5.13	4.05
AF-membranes-DC	2.89	6.14	4.02
AF-p-membranes	3.42	7.15	4.83
AF-p-membranes-DC	3.71	15.75	7.01

**Table 2:** Diffusion rate of a water droplet on the PVDF/CFO membranes.

	AF-membranes	AF-membranes-DC	AF-p-membranes	AF-p-membranes-DC
**Diffusion rate** **(cm^2^/s)**	0.23	0.15	0.11	0.05
